# Supplementary material for: Prevalence and Resistance Profiles of Acinetobacter baumannii in ICU Patients in Saudi Arabia: A Systematic Review and Meta-Analysis
Source: Antibiotics (Basel). 2025 Nov 7;14(11):1131. doi: 10.3390/antibiotics14111131 (PMC12649208; doi:10.3390/antibiotics14111131)
Supplement: Supplementary file 1 [file antibiotics-14-01131-s001.zip › Supplementary Table (Table S3) - PRISMA Algorithm.pdf]

Supplementary Table S3. Full Electronic Search Strategies Used in Each Database

Database	Search Strategy (exact algorithm)	Limits / Filters	Last Search Date
PubMed	("Acinetobacter baumannii"[Mesh] OR "Acinetobacter baumannii" OR "A. baumannii") AND ("multidrug resistant" OR MDR OR "drug resistance" OR "carbapenem-resistant" OR CRAB) AND ("intensive care units"[Mesh] OR "intensive care unit" OR ICU OR "critical care") AND ("Saudi Arabia"[Mesh] OR "Saudi Arabia")	Humans; English; 2014–2025	30 September 2025
Scopus	TITLE-ABS-KEY("Acinetobacter baumannii") AND TITLE-ABS-KEY("multidrug resistant" OR MDR OR "carbapenem resistant" OR CRAB) AND TITLE-ABS-KEY("ICU" OR "intensive care" OR "critical care") AND TITLE-ABS-KEY("Saudi Arabia")	English; 2014–2025; Article types: Article	30 September 2025
Web of Science Core Collection	TS=("Acinetobacter baumannii") AND TS=("multidrug resistant" OR MDR OR "carbapenem resistant" OR CRAB) AND TS=("ICU" OR "intensive care" OR "critical care") AND TS=("Saudi Arabia")	English; 2014–2025; Document types: Article	30 September 2025
Saudi Digital Library (SDL)	"Acinetobacter baumannii" AND ("ICU" OR "intensive care" OR "critical care") AND "Saudi Arabia"	English; 2014–2025	30 September 2025
