# Supplementary material for: Prevalence and Resistance Profiles of Acinetobacter baumannii in ICU Patients in Saudi Arabia: A Systematic Review and Meta-Analysis
Source: Antibiotics (Basel). 2025 Nov 7;14(11):1131. doi: 10.3390/antibiotics14111131 (PMC12649208; doi:10.3390/antibiotics14111131)
Supplement: Supplementary file 1 [file antibiotics-14-01131-s001.zip › Supplementary Table (Table S4)-JBI.pdf]

Supplementary Table (Table S4). Quality Assessment of Included Studies using the JBI Checklist

Study	Inclusion criteria	Study subject and settings	Exposure measurement	Objectives/standard criteria	Confounders identified	Confounders control	Outcome measurement	Statistical tests	Risk category
Alotaibi et al. 2021. (27)	*	*	*	*	*	*	*	*	Low
Al-Omari et al. 2017. (28)	*	*	*	*	*	*	*	*	Low
Aljindan et al. 2015 (36)	*	*	*	*	*	*	*	*	Low
Kharaba et al. 2021 (37)	*	*	*	*	*	*	*	*	Low
Al Bshabshe et al. 2016 (29)	*	*	*	*	*	*	*	*	Low
Alhaddad et al. 2018. (38)	*	*	*	*	*	*	*	*	Low
Al-Sultan. 2021 (39)	*	*	*	*	*	*	*	*	Low
Hafiz et al. 2023. (30)	*	*	*	*	*	*	*	*	Low
Almaghrabi et al. 2018 (40)	*	*	*	*	*	*	*	*	Low
Aedh et al. 2023. (41)	*	*	*	*	*	*	*	*	Low
Al-Otaibi et al. 2016. (31)	*	*	*	*	–	–	*	*	Medium
Al-Obeid et al. 2015. (32)	*	*	*	*	*	*	*	*	Low
Alharbi et al. 2025. (34)	*	*	*	*	*	*	*	*	Low
Mwanri L & AlSaleh E. 2014. (42)	*	*	*	*	–	–	*	*	Medium
Gaifer et al. 2024. (35)	*	*	*	*	*	*	*	*	Low
Kaki R. 2024. (33)	*	*	*	*	*	*	*	*	Low
Alanazi FA et al. 2025 (43)	*	*	*	*	*	*	*	*	Low

** Medium risk of bias studies. Remaining studies had low risk of bias.
